# Transient Receptor Potential (TRP) and Cch1-Yam8 Channels Play Key Roles in the Regulation of Cytoplasmic Ca^2+^ in Fission Yeast

**DOI:** 10.1371/journal.pone.0022421

**Published:** 2011-07-19

**Authors:** Yan Ma, Reiko Sugiura, Atsushi Koike, Hidemine Ebina, Susie O. Sio, Takayoshi Kuno

**Affiliations:** 1 Division of Molecular Pharmacology and Pharmacogenomics, Department of Biochemistry and Molecular Biology, Kobe University Graduate School of Medicine, Kobe, Japan; 2 Laboratory of Molecular Pharmacogenomics, School of Pharmaceutical Sciences, Kinki University, Higashi-Osaka, Japan; 3 Department of Pharmacology and Toxicology, College of Medicine, University of the Philippines Manila, Manila, Philippines; University of Cambridge, United Kingdom

## Abstract

The regulation of cytoplasmic Ca^2+^ is crucial for various cellular processes. Here, we examined the cytoplasmic Ca^2+^ levels in living fission yeast cells by a highly sensitive bioluminescence resonance energy transfer-based assay using GFP-aequorin fusion protein linked by 19 amino acid. We monitored the cytoplasmic Ca^2+^ level and its change caused by extracellular stimulants such as CaCl_2_ or NaCl plus FK506 (calcineurin inhibitor). We found that the extracellularly added Ca^2+^ caused a dose-dependent increase in the cytoplasmic Ca^2+^ level and resulted in a burst-like peak. The overexpression of two transient receptor potential (TRP) channel homologues, Trp1322 or Pkd2, markedly enhanced this response. Interestingly, the burst-like peak upon TRP overexpression was completely abolished by gene deletion of calcineurin and was dramatically decreased by gene deletion of Prz1, a downstream transcription factor activated by calcineurin. Furthermore, 1 hour treatment with FK506 failed to suppress the burst-like peak. These results suggest that the burst-like Ca^2+^ peak is dependent on the transcriptional activity of Prz1, but not on the direct TRP dephosphorylation. We also found that extracellularly added NaCl plus FK506 caused a synergistic cytosolic Ca^2+^ increase that is dependent on the inhibition of calcineurin activity, but not on the inhibition of Prz1. The synergistic Ca^2+^ increase is abolished by the addition of the Ca^2+^ chelator BAPTA into the media, and is also abolished by deletion of the gene encoding a subunit of the Cch1-Yam8 Ca^2+^ channel complex, indicating that the synergistic increase is caused by the Ca^2+^ influx from the extracellular medium via the Cch1-Yam8 complex. Furthermore, deletion of Pmk1 MAPK abolished the Ca^2+^ influx, and overexpression of the constitutively active Pek1 MAPKK enhanced the influx. These results suggest that Pmk1 MAPK and calcineurin positively and negatively regulate the Cch1-Yam8 complex, respectively, via modulating the balance between phosphorylation and dyphosphorylation state.

## Introduction

Calcium signaling is an important regulator in all eukaryotic cells for a wide variety of physiological processes. The cells have evolved mechanisms to regulate cytoplasmic Ca^2+^ homeostasis in response to external or internal stress. Small changes in cytoplasmic Ca^2+^ levels can activate various Ca^2+^-sensing proteins, such as calmodulin and calcineurin, which then lead to the induction of various downstream signal transduction pathways. In response to external or internal stress the cells have evolved mechanisms to regulate cytoplasmic Ca^2+^ homeostasis. In mammalian cells, various channels including the voltage-gated calcium channels and the transient receptor potential (TRP) channels play important roles in regulating cytoplasmic Ca^2+^ [1]–[3]. In budding yeast *Saccharomyces cerevisiae*, vacuolar TRP channel Yvc1p mediates the release of Ca^2+^ from the vacuole in response to hyperosmotic shock [4], [5], and Cch1p and Mid1p form a mechano-sensitive channel complex for Ca^2+^ influx at the plasma membrane [6]. In fission yeast *Schizosaccharomyces pombe*, the TRP channel Pkd2 plays important roles in cell wall synthesis and membrane trafficking [7], [8], and Yam8/Ehs1 is involved in maintaining cell wall integrity and in calcium uptake [9].

In our previous study, we monitored the cytoplasmic Ca^2+^ levels for 10 min using aequorin (AEQ) in living fission yeast cells transformed with pREP1-AEQ [10]. However, it was necessary to concentrate the cells to improve the sensitivity of the measurement, and the increased cell concentration makes it unsuitable for monitoring the Ca^2+^ levels over a long period of time. It was reported that a GFP-AEQ fusion protein linked by 19 amino acid (GFP-19-AEQ) closely approximates the *Aequorea victoria* jellyfish GFP and results in an efficient bioluminescence resonance energy transfer to provide a highly sensitive Ca^2+^ sensor [11]. In the present study, we used the GFP-19-AEQ fusion protein to monitor the cytoplasmic Ca^2+^ level in living fission yeast cells. By monitoring cytoplasmic Ca^2+^ under various conditions, we showed that two TRP homologues, Trp1322 and Pkd2, mediate the cytoplasmic Ca^2+^ rise caused by the extracellularly added CaCl_2_, and that the Cch1-Yam8 channel complex mediates the cytoplasmic Ca^2+^ rise caused by the extracellularly added NaCl plus FK506. Also, we showed that calcineurin play important roles in these two processes.

## Materials and Methods

### Strains, Media, and Genetic and Molecular Biology Methods

*S. pombe* strains used in this study are listed in Table 1. The complete medium YPD (yeast extract-peptone-dextrose) and the minimal medium EMM (Edinburgh minimal medium) have been described previously [12]. Standard genetic and recombinant-DNA methods [13] are used except where noted. FK506 was provided by Astellas Pharma Inc. (Japan). Gene disruptions are denoted by lowercase letters representing the disrupted gene followed by two colons and the wild-type gene marker used for disruption (for example, *trp1322*::*ura4* ^+^). Gene disruptions are abbreviated by the gene preceded by Δ (for example, Δ*trp1322*). Proteins are denoted by Roman letters and only the first letter is capitalized (for example, Trp1322).

**Table 1:** Strains used in this study.

Strain	Genotype	Reference
HM123	*h^−^ leu1-32*	Our stock
KP456	*h^−^ leu1-32 ura4-D18*	Our stock
HM528	*h^+^ his2*	Our stock
KP928	*h^+^ his2 leu1-32 ura4-D18*	Our stock
KP119	*h^+^ leu1-32 ura4-D18 ppb1:: ura4^+^*	Our stock
KP1248	*h* ^−^ *leu1-32 ura4-294*	Our stock
KP2101	*h^−^ leu1-32 arg1-1*	[18]
KP1003	*h^−^ leu1-32 ura4-D18 prz1:: ura4^+^*	[28]
KP2784	*h^−^ leu1-32 ura4-D18 cch1:: ura4^+^*	[10]
KP 208	*h^−^ leu1-32 ura4-D18 pmk1:: ura4^+^*	[36]
KP3025	*h^−^ leu1-32 ura4-D18 pmr1::ura4^+^ arg1-1 pREP1-GFP-19aa-AEQ*::*arg1^+^*	This study
KP3028	*h^−^ leu1-32 arg1-1 pREP1-GFP-19aa-AEQ*::*arg1^+^*	This study
KP3366	*h^−^ leu1-32 ura4-D18 trp1322:: ura4^+^*	This study
KP3381	*h^−^ leu1-32 arg1-1 nmt1 GST-Pek1^DD^*::*arg1^+^*	This study
KP4253	*h^−^ leu1-32 arg1-1 ura4D-18 trp1322*::*ura4^+^ nmt1GST-Pek1^DD^*::*arg1^+^*	This study
KP2755	*h^−^ leu1 arg1* 3×CDRE::luc(R2.2)::*arg1^+^*	This study
KP3750	*h^−^ leu1-32 arg1-1 ura4 D-18 ppb1*::*ura4^+^ pREP1-GFP-19aa-AEQ*::*arg1^+^*	This study
KP3688	*h^−^ leu1-32 arg1-1 ura4 D-18 prz1*::*ura4^+^ pREP1-GFP-19aa-AEQ*::*arg1^+^*	This study
KP2770	*h− leu1 ura4D-18 yam8*::*KanMX_6_*	This study
KP3334	*h− leu1 ura4D-18 yam8*::*KanMX_6_ cch1*:: *ura4^+^*	This study
KP5088	*h^−^ leu1-32 ura4-294 arg1-1 pREP1-Yam8-GFP pREP1-GFP-19aa-AEQ*::*arg1^+^*	This study
KP5059	*h^−^ leu1 ura4 cam1-116F≪ura4^+^*	[37]

### Gene Expression

For ectopic expression of proteins, the thiamine-repressible *nmt1* promoter [14] and the constitutive *adh1* promoter [15] were used. The expression vector pREP41-GFP-19-AEQ was constructed in two steps. First, two oligonucleotides, 5^′^-GAT CTC CCG GTA CTG CCA CTC CCG CCA CTA CTC CCA CTA CTG CCC CCA CTG CCG GTA CTG GTC TGC AGG GAT CCT GC-3^′^ and 5^′^-GCG GCC GCA GGA TCC CTG CAG ACC AGT ACC GGC AGT GGG GGC AGT AGT GGG AGT AGT GGC GGG AGT GGC AGT ACC GGG A-3^′^ , were mixed in equimolar amounts, reannealed, and cloned into the *Bam*HI/*Not*I site of the pREP1-N-GFP vector (pKB4663) to give pREP1-GFP-19 amino acid (pKB5995). Second, the apoaequorin (AEQ) gene was amplified by polymerase chain reaction (PCR) from the pEVP11/AEQ plasmid [16] and ligated to pKB5995 to give pREP1-GFP-19-AEQ (pKB6011). The *Xho*I/*Not*I fragment containing GFP-19-AEQ was subcloned into the *Xho*I/*Not*I pKB1894 (pREP41-N-GST) to give pREP41-GFP-19-AEQ (pKB6045).

The *adh1* promoter was amplified by PCR using the pART1 vector containing an *adh1* promoter [15] as a template and using the sense primer 5^′^-CGC GGA TCC TGC AGT GCA TGC CCT ACA ACA ACT AAG-3^′^ , and the antisense primer 5^′^-CGC GGA TCC TCG AGG AAT TCT CTT GCT TAA AGA AAA G-3^′^ . The amplified product containing the *adh1* promoter was digested with *Bam*HI, and the resulting fragment was subcloned into BlueScriptSK (+) (Stratagene) to give pKB6881. Then the *Pst*I/*Xho*I fragment of pKB6881 was ligated into the *Pst*I/*Xho*I site of pKB1894 to give pKB6891. Finally, the above-mentioned *Xho*I/*Not*I fragment containing GFP-19-AEQ from pKB6011 was ligated into *Xho*I/*Not*I site of pKB6891 to give *adh1*-GFP-19-AEQ (pKB6892).

The constitutively active Pmk1 MAPKK Pek1^DD^ [17] was chromosomally expressed as follows. The *Pst*I/*Sac*I fragment containing pREP1-GST-Pek1^DD^ was subcloned into the *Pst*I/*Sac*I site of pKB5049 bearing the *arg1* ^+^ marker [18]. The resulting construct was digested with *Stu*I, and transformed into KP2101 cells [18]. Stable integrants were selected on medium lacking arginine, and integration was checked by genomic Southern hybridization (data not shown). The expression was repressed by the addition of 4 µM thiamine to EMM, and was induced by washing and incubating the cells in EMM lacking thiamine for 24 hours.

### Measurement and Quantification of Cytoplasmic Ca^2+^ Levels Using GFP-19-AEQ

The cytoplasmic Ca^2+^ levels were determined using a previously described method with minor modifications [10]. In brief, cells expressing GFP-19-AEQ were resuspended in fresh EMM containing 5 µM coelenterazine (Promega, catalog #S200A), and the optical density was adjusted to 0.3 at 660 nm. To convert apoaequorin to aequorin, the cells were incubated for 4 hours at 27°C. The cells were washed twice, resuspended in fresh EMM and the optical density was adjusted to 0.6 at 660 nm. Then the cell culture was incubated at 27°C for 30 min before initiating the experiment. The light emission levels expressed as relative light units (RLU) were measured using a luminometer. For the quantification of the molar concentration of the cytosolic Ca^2+^ level, a method previously described was used with minor modifications [19]. In brief, the luminescence from aequorin that remained in cells at the end of an experiment was determined after treating the cells with 7.5% Triton X-100 and 2 M CaCl_2_.

### Tagging and Deletion of the *trp1322^+^* (SPCC1322.03c), *trp663^+^* (SPCC663.14), and *pkd2^+^* Genes

The three TRP genes were amplified by PCR with the genomic DNA of wild-type cells as a template. For the *trp1322^+^* gene (SPCC1322.03), the sense primer used was 5′-GGA AGA TCT ATG AAG CCT CGC CCG TAC TCT G-3′, and the antisense primer was 5′-GGA AGA TCT TTA TGG ATG TGG AAT CGA CC-3′. For the *trp663^+^* gene (SPCC663.14c), the sense primer used was 5′-GGA AGA TCT ATG AAG CTA ATA CTC TTA GC-3′, and the antisense primer was 5′-GGA AGA TCT CGA ACA GTA CGC TGC ATT TCA TTA ATC CCG-3′. For the *pkd2^+^* gene, the sense primer used was 5′-CGC GGA TCC CAT ATG AGG CTT TGG AGA AGC CC-3′, and the antisense primer was 5′-CGC GGA TCC CGA CGA AAA GCA TTG TTA GGT AAT GG-3′. The amplified product containing the *trp1322^+^* or *trp663^+^* gene was digested with *Bgl*II, and the resulting fragment was subcloned into BlueScriptSK (+) (Stratagene) in which *Bam*HI site was changed to a *Bgl*II site. The amplified product containing the *pkd2^+^* gene was digested with *Bam*HI, and the resulting fragment was subcloned into BlueScriptSK (+). As described previously [17], [10], to express GFP-Trp1322, the complete open reading frame (ORF) of *trp1322^+^* was amplified by PCR and was ligated to the N-terminus of the GFP carrying the S65T mutation [20]. To express Pkd2-GFP or Trp663-GFP, the complete ORF of *pkd2^+^* or *trp663^+^* was amplified by PCR and was ligated to the C-terminus of the GFP carrying the S65T mutation [20].

To knockout the *trp1322^+^* gene (SPCC1322.03), a one-step gene disruption by homologous recombination was performed [21]. The *trp1322*::*ura4^+^* disruption was constructed as follows. The *Bgl*II fragment containing the ORF of the *trp1322^+^* gene was subcloned into BlueScriptSK (+) in which *Bam*HI site was changed to a *Bgl*II site. Then, a *Bam*HI fragment containing the *ura4^+^* gene was inserted into the *Bam*HI site of the previous construct. The fragment containing the disrupted *trp1322^+^* gene was transformed into diploid cells. Stable integrants were selected on medium lacking uracil. The disruption of the gene was checked by genomic Southern hybridization (data not shown).

### Deletion of the *yam8^+^* Gene Using a *KanMX*_6_ Marker

The *yam8* ^+^ gene was disrupted by the insertion of *KanMX*_6_ cassette at the *Bam*HI site of the Yam8 ORF. The *yam8*::*KanMX_6_* cells were constructed using a previously described method with minor modifications [22]. Briefly, the fragments containing the disrupted *yam8* gene were transformed into KP456 cells. The stable integrants were selected on G418 plates (100 mg/l), and disruption of the gene was checked by genomic Southern hybridization (data not shown).

### DAPI Staining

For DAPI staining, the cells grown to log-phase were fixed for 10 min in a solution of 3.7% formaldehyde and washed twice in 1×PBS, and then were resuspended in 100 µl 1×PBS. Suitable quantities of the cells were stained with DAPI for 10 min, and then were observed under the microscope.

### Microscopy and Miscellaneous Methods

Methods in light microscopy, such as differential interference contrast (DIC) and fluorescence microscopy, that were used to observe the GFP-19-AEQ were performed as described [23]. Cell extract preparation and immunoblot analysis were performed as described [24]. Calcineurin-dependent response element (CDRE)-reporter activity was measured as described [10].

## Results

### Real-time monitoring of the cytoplasmic Ca^2+^ levels using GFP-19-AEQ in fission yeast

**Figure 1:**
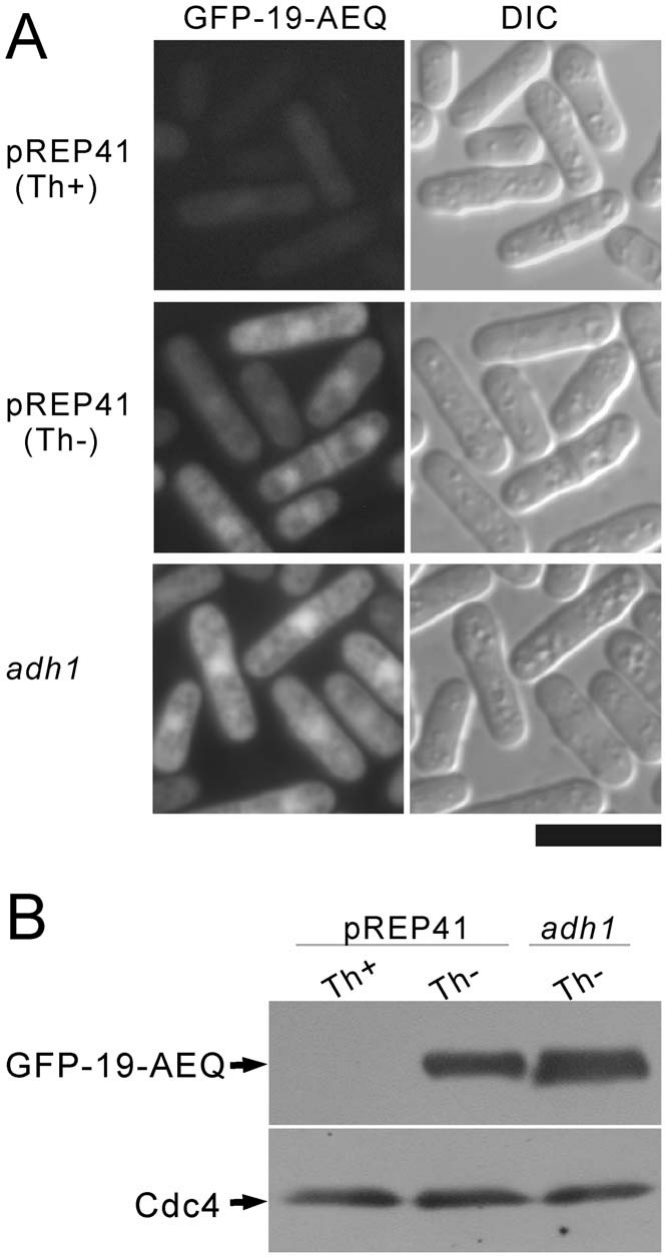
Fluorescence microscopy and immunoblot analysis of GFP-19-AEQ. (A) Fluorescence imaging of GFP-19-AEQ. The pREP41-GFP-19-AEQ (pKB6045) or adh1-GFP-19-AEQ (pKB6892) was transformed into the wild-type cells. The transformants were grown to early log phase in EMM with (Th+) or without (Th-) 4 µM thiamine to repress and to induce the expression of GFP-19-AEQ, respectively. Then, the cells were examined under the fluorescence microscope (GFP-19-AEQ) and the differential interference contrast microscope (DIC) as described in Materials and Methods. Bar, 10 µm. (B) Immunoblot analysis of GFP-19-AEQ. The wild-type cells harboring pREP41-GFP-19-AEQ (pKB6045) or *adh1*-GFP-19-AEQ (pKB6892) were cultured as described in Figure 1A, then the cell extracts were subjected to electrophoresis using 10% polyacrylamide gel and were immunoblotted using-GFP antibodies to detect GFP-19-AEQ, or subjected to electrophoresis using 15% polyacrylamide gel and were immunoblotted using anti-Cdc4 antibodies to detect endogenous Cdc4 (loading control).

For real-time monitoring of the cytoplasmic Ca^2+^ levels in fission yeast, we expressed GFP-19-AEQ [11] in wild-type cells under the control of the *adh1* promoter or the thiamine-repressible *nmt1* promoter, and the fluorescence was checked by microscopy. Under the *nmt1* promoter of the strong expression vector pREP1, GFP-19-AEQ aggregated as dots in the cells (data not shown). Under the *nmt1* promoter of the attenuated expression vector pREP41, the fluorescence of GFP-19-AEQ localized to both the cytoplasm and nucleus (Figure 1A, middle panel), and the fluorescence became very weak under repressing conditions (Figure 1A, upper panel). Under the *adh1* promoter, the fluorescence was a little stronger (Figure 1A, lower panel) than the *nmt1* promoter using pREP41 under inducing conditions (Figure 1A, middle panel). The GFP-19-AEQ fusion protein migrated as a single band corresponding to the calculated molecular mass of about 50 kDa in SDS-PAGE gel (Figure 1B), and the expression level was consistent with the strength of the fluorescence (Figure 1A).

**Figure 2:**
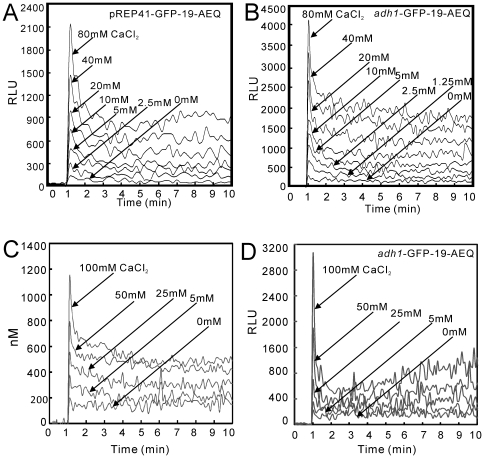
Real-time monitoring of the cytoplasmic Ca^2+^ level using GFP-19-AEQ. Monitoring of the cytoplasmic Ca^2+^ level using pREP41-GFP-19-AEQ (A) and *adh1*-GFP-19-AEQ reporter vector (B). The wild-type cells were transformed with pREP41-GFP-19-AEQ (pKB6045) or *adh1*-GFP-19-AEQ (pKB6892). The transformants were grown to exponential phase in the absence of thiamine for 36 hours to express apoaequorin. Then, the cells were collected and treated as described in Materials and Methods. A 10 µl volume of distilled water or 10× stock of various concentration of CaCl_2_ were added into the 96-well plate, and the cells were delivered to the wells via the luminometer pump, and the luminescence was followed for 10 min. The aequorin luminescence, given as relative light units (RLU) s^−1^, is plotted versus time. The data shown are representative of multiple experiments. (C) The molar concentration of the cytosolic Ca^2+^ level in wild-type cells. The wild-type cells harboring reporter vector pKB6892 were cultured and assayed as described in Figure 2B. Then the quantification of cytoplasmic Ca^2+^ concentration was performed as described in Materials and Methods. The data shown are representative of multiple experiments. (D) The burst-like peak seen in wild-type cells was similarly observed in the *cam1-1* mutant cells. The *cam1-1* mutant cells harboring reporter vector pKB6892 were cultured and assayed as described in Figure 2B. The data shown are representative of multiple experiments.

The wild-type cells harboring *nmt41*-GFP-19-AEQ or *adh1*-GFP-19-AEQ were cultured as described in Materials and Methods, and then the cells were stimulated by the addition of various concentrations of extracellular CaCl_2_ (0 mM to 80 mM). As shown in Figure 2, upon the addition of CaCl_2_, the cytoplasmic Ca^2+^ level increased immediately and reached a peak level, and then decreased to a new steady-state within 2–3 min in a dose-dependent manner. The steady-state was sustained for at least 4 hours (data not shown). This pattern suggests a markedly improved sensitivity over our previous report using pREP1-AEQ and is consistent with the pattern of CDRE-reporter assay [10]. The sensitivity using *adh1*-GFP-19-AEQ (Figure 2B) was higher than that using the *nmt41*-GFP-19-AEQ (Figure 2A). Thus, the *adh1*-GFP-19-AEQ reporter assay system was selected. Furthermore, we determined the molar concentration of the cytosolic Ca^2+^ level as described in Materials and Methods. The results showed that the resting cytosolic Ca^2+^ concentration in fission yeast varies from 100∼200 nM, and the stimulation by extracellularly added 100 mM CaCl_2_ induced an approximately 6∼10 fold increase at the peak of the burst (Figure 2C).

To investigate whether the Ca^2+^ sensor proteins like calmodulin or Ncs1 are responsible for the Ca^2+^-induced channel inactivation similar to what is found in mammalian Ca^2+^ channels [25], [26], we monitored the cytosolic Ca^2+^ concentration in several mutant cells. In *cam1-1* mutant cells, as shown in Figure 2D, the burst-like peak seen in wild-type cells was also observed. In Δ*ncs1* cells the burst-like peak seen in wild-type cells was similarly observed (data not shown).

### Transient receptor potential (TRP) channels, Trp1322 and Pkd2, are Ca^2+^-permeable and play roles in the extracellularly added CaCl_2_-induced cytoplasmic Ca^2+^ increase

**Figure 3:**
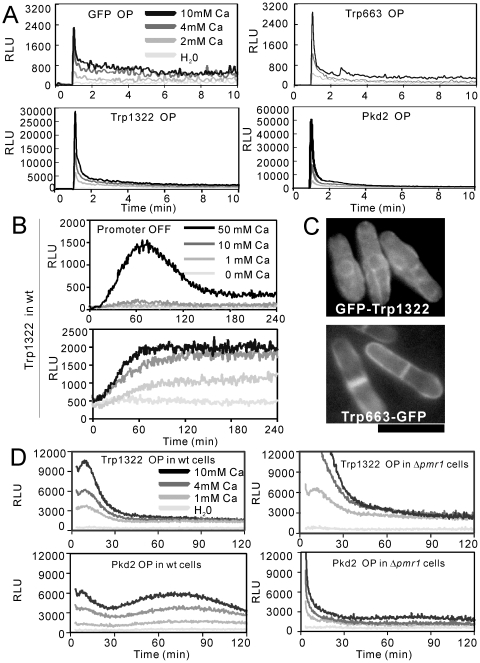
TRP channels play key roles in the regulation of cytoplasmic Ca^2+^ in fission yeast. (A) The overexpression of *trp1322* ^+^ and *pkd2* ^+^ markedly enhanced the response to extracellularly added Ca^2+^. The KP3028 cells were transformed with a control vector, pREP1-GFP-Trp1322, or pREP1-Pkd2-GFP. The transformants were grown to exponential phase in the absence of thiamine for 36 hours to express GFP-19-AEQ, Trp1322 or Pkd2. The monitoring of the cytoplasmic Ca^2+^ level was performed as described in Figure 2A. The data shown are representative of multiple experiments. (B) The CDRE-reporter activity in the cells overexpressing Trp1322. The KP2755 (*h^−^ leu1 arg1* 3×CDRE::luc(R2.2)::*arg1^+^*) cells were transformed with pREP1-GFP-Trp1322. The transformants were grown to exponential phase in the presence (Promoter OFF) or absence (Promoter ON, overexpression) of thiamine for 20 hours. The CDRE assay was performed as described in Materials and Methods. The data shown are representative of multiple experiments. (C) Intracellular localization of the two TRP channels. The wild-type cells harboring GFP-Trp1322 or Trp663-GFP were grown to early log phase in EMM containing 4 µM thiamine, and then were examined under the fluorescence microscope. Bar, 10 µm. (D) Pkd2 plays roles in inducing Ca^2+^ release from the Golgi. The KP3028 or KP3025 cells were transformed with pREP1-GFP-Trp1322 or pREP1-Pkd2-GFP, then were cultured and assayed as described in Figure 2A.

The TRP channel superfamily consists of a diverse group of cation channels, and plays critical roles in response to external stimuli [1]–[3]. Blast searches against Sanger Center *S. pombe* databases led to the identification of three open reading frames which are as follows, SPCC1322.03 (named as *trp1322^+^* in this study), SPCC663.14c (named as *trp663^+^* in this study), and SPAC1F7.03 (Pkd2) [8] that encode TRP-like ion channels. We subcloned each of the three TRP channels in pREP1 vector and examined the effect of their overexpression on the cytoplasmic Ca^2+^ levels in wild-type cells. The results showed that the overexpression of Trp1322 or Pkd2 markedly induced an approximately 10∼20 fold burst-like increase in the cytoplasmic Ca^2+^ levels when stimulated by the extracellularly added CaCl_2_ as compared with the control vector (Figure 3A). In contrast, the overexpression of Trp663 induced a cytoplasmic Ca^2+^ increase similar to that of the control vector (Figure 3A).

To investigate whether the increased cytoplasmic Ca^2+^ activates calcineurin, we monitored the CDRE-reporter activity [10]. Results showed that the basal activity was very high and a near maximal response was elicited by the extracellular addition of 10 mM CaCl_2_ (Figure 3B). Also, the overexpression of Pkd2 induced a very high CDRE response and a near maximal response was elicited by the extracellular addition of 10 mM CaCl_2_ (data not shown).

We then observed the intracellular localization of the three TRP homologues. GFP-Pkd2 localized to the membrane structures as described previously by other investigators (data not shown) [7], [8]. As shown in Figure 3C, overexpressed GFP-Trp1322 mainly localize to the intracellular membrane structures, while overexpressed Trp663-GFP clearly localized to the plasma membrane (Figure 3C). We tried to confirm the localization of Trp1322 and Trp663 by using integrated GFP constructs under endogenous promoters as follows. Regarding Trp1322, the C-terminal tagged protein is non-functional (data not shown), and chromosomally integrated GFP-Trp1322 under *nmt1* promoter did not show any clear localization signals in the presence of thiamine, and showed a similar localization to that of the plasmid-based GFP-Trp1322 in the absence of thiamine (data not shown). Regarding Trp633, a strain with the integrated GFP constructs under endogenous promoters was made, but no signals were observed (data not shown), suggesting that Trp633 did not show any clear localization signals when produced under physiological conditions.

### Pkd2 may mediate Ca^2+^ release from the Golgi

As shown in Figure 3D, the overexpression of Pkd2, but not Trp1322, exhibited a less pronounced second peak in the cytoplasmic Ca^2+^ level at 60 to 90 min after the addition of CaCl_2_, suggesting that Pkd2 channel may be responsible for the release of Ca^2+^ from the intracellular organelles. To investigate whether the Golgi or the vacuole is responsible for the biphasic increase, we monitored the effect of Pkd2 overexpression in the knockout cells of Pmr1 (Golgi membrane Ca^2+^ pump), Pmc1 (vacuolar Ca^2+^ ATPase), and Vcx1 (vacuolar H^+^/Ca^2+^ exchanger). In Δ*pmr1*, Δ*pmc1* and Δ*vcx1* cells respectively, the burst-like first peaks were also observed (data not shown). Interestingly, in Δ*pmr1* cells the second peak was not observed (Figure 3D), suggesting that the increased cytoplasmic Ca^2+^ level in wild-type cells overexpressing Pkd2 is derived at least, in part, from the Golgi.

### NaCl plus FK506 treatment caused a slow and persistent increase in the cytoplasmic Ca^2+^ level

**Figure 4:**
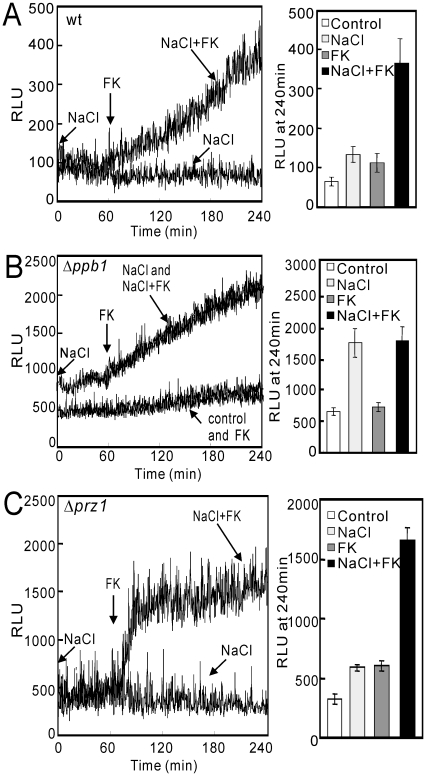
NaCl plus FK506 treatment caused a slow and persistent increase in the cytoplasmic Ca^2+^ level. The wild-type (A), Δ*ppb1* (B) or Δ*prz1* (C) cells harboring pKB6892 were cultured as described in Figure 2B. A 10 µl volume of distilled water or 2 M stock of NaCl were added into the 96-well plate, and then 90 µl volume of cells were rapidly added to each sample. The cells were incubated for 30 min to reach a steady-state cytoplasmic Ca^2+^ level and the luminescence was followed for 60 min. Then 2 µl of distilled water or FK506 with a concentration of 50 µg/ml were added to the samples and the luminescence was monitored up to 240 min. Left panel: The data shown are representative of multiple experiments. Right panel: The data represent the means ± standard deviations of RLU taken at 240 min from three independent experiments, and each sample was analyzed in duplicate.

Our previous result showed that high extracellular NaCl concentration caused an increase in the cytoplasmic Ca^2+^ level and also elicited the stimulation of the CDRE-reporter activity in fission yeast [10]. To uncover the mechanism of the NaCl-induced calcium increase and to investigate the role of calcineurin, we monitored the cytoplasmic Ca^2+^ level upon the addition of NaCl, FK506 (a specific inhibitor of calcineurin), or NaCl plus FK506. Wild-type cells expressing GFP-19-AEQ were cultured as described in Materials and Methods. Interestingly, compared to the cells treated with NaCl alone or FK506 alone, the addition of FK506 to the NaCl pretreated cells caused a slow and persistent increase in the cytoplasmic Ca^2+^ level in wild-type cells (Figure 4A, left panel). The addition of extracellular NaCl alone and FK506 alone induced an approximately 2.4-fold and 1.8-fold increase in relative light units (RLU), respectively (Figure 4A, right panel). The addition of NaCl plus FK506 induced an approximately 6-fold increase after treatment with the agents for 4 hours (Figure 4A, right panel). The persistent rise in the Ca^2+^ level was observed for at least 6 hours and the Ca^2+^ elevation continued on until all aequorin was consumed (data not shown).

### The synergistic increase in the cytoplasmic Ca^2+^ level depends on the inhibition of calcineurin activity, but not on the inhibition of Prz1 transcriptional activity

To determine whether the synergistic increase in the cytoplasmic Ca^2+^ level was caused by the inhibition of calcineurin activity, we monitored the level of cytoplasmic Ca^2+^ in the knockout cells of the *ppb1* ^+^ gene, encoding a single catalytic subunit of fission yeast calcineurin [27]. In Δ*ppb1* cells, the extracellularly added NaCl caused a persistent increase in the cytoplasmic Ca^2+^ level, and the addition of FK506 did not cause any further increase (Figure 4B), indicating that the synergistic increase in the cytoplasmic Ca^2+^ level is caused by the inhibition of calcineurin. It should be noted that in Δ*ppb1* cells (Figure 4B, control), the basal RLU was significantly higher than that in the wild-type cells (Figure 4A, control).

Our previous study showed that calcineurin dephosphorylates and activates the Prz1 transcription factor in fission yeast [28]. This prompted us to monitor the level of cytoplasmic Ca^2+^ in Δ*prz1* cells. As shown in Figure 4C in Δ*prz1* cells, the synergistic increase in the cytoplasmic Ca^2+^ level was clearly observed suggesting that the increase is not mediated by Prz1. Notably in Δ*prz1* cells, NaCl plus FK506 induced a rapid and sharp increase in the Ca^2+^ level (Figure 4C, left panel), whereas in wild-type cells the same treatment induced a slow and persistent increase (Figure 4A, left panel). We speculate that Prz1 might possess some unknown function in regulating calcium homeostasis in addition to its transcriptional activity.

### The synergistic increase in the cytoplasmic Ca^2+^ level is due to Ca^2+^ influx from the extracellular medium via the Cch1-Yam8 channel complex

**Figure 5:**
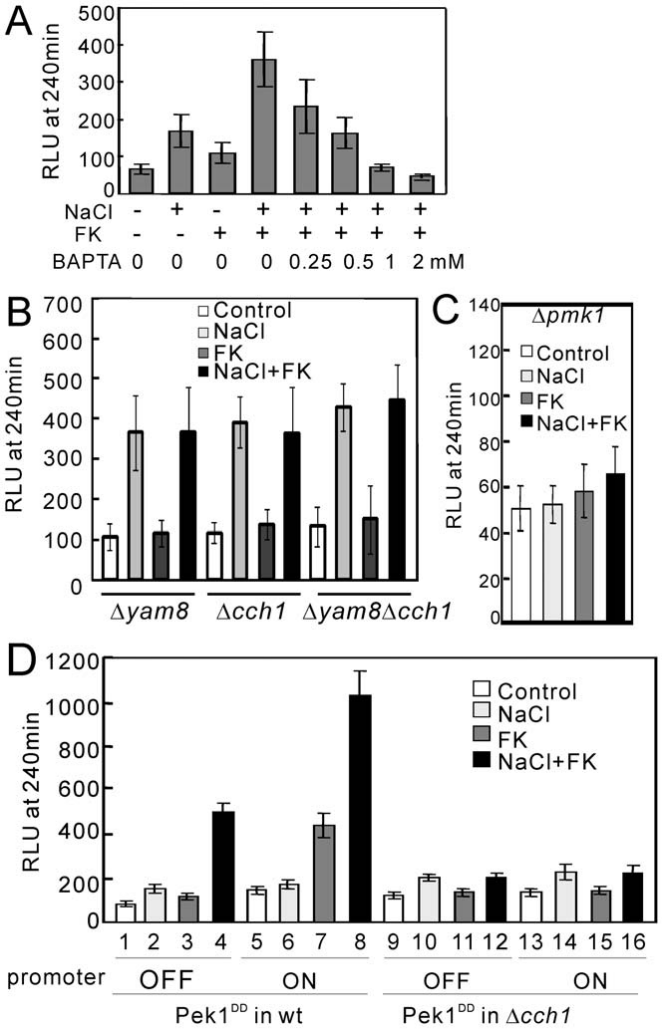
NaCl plus FK506 caused a synergistic cytoplasmic Ca^2+^ increase via the Cch1-Yam8 channel complex. (A) The synergistic increase in the cytoplasmic Ca^2+^ level is derived from the extracellular medium. The experiment was performed as described in Figure 4, except that prior to the addition of FK506, various concentrations of BAPTA (0.25, 0.5, 1, and 2 mM) were added to chelate Ca^2+^ in the EMM medium. The histogram was calculated as described in the legend of Figure 4. (B) The *cch1* and *yam8* deletion abolished the synergistic increase in the cytoplasmic Ca^2+^ level. The Δ*yam8*, Δ*cch1*, or Δ*yam8*Δ*cch1* cells harboring pKB6892 were cultured and assayed as described in Figure 4. The histogram was calculated as described in the legend of Figure 4. (C) The *pmk1* deletion abolished the synergistic increase in the cytoplasmic Ca^2+^ level. The Δ*pmk1* cells harboring pKB6892 were cultured and assayed as described in Figure 4. The histogram was calculated as described in the legend of Figure 4. (D) Overexpression of the constitutively active Pek1 MAPKK stimulates the Cch1-Yam8-mediated Ca^2+^ influx. The wild-type or Δ*cch1* cells integrated with chromosomal pREP1-GST-Pek1^DD^ were transformed with pKB6892, and the transformants were cultured in EMM containing 4 µM thiamine for 12 hours. Then the cells were collected and washed three times with EMM without thiamine. The washed cells were divided into two portions, one portion was cultured in EMM containing 4 µM thiamine, and the other portion was cultured in EMM without thiamine, and the cells were grown to exponential phase. The cytoplasmic Ca^2+^ level was monitored as described in Figure 4. “Promoter OFF” indicates that the expression of Pek1^DD^ was repressed by the addition of 4 µM thiamine to the medium and “Promoter ON” indicates that the expression of Pek1^DD^ was induced by the removal of thiamine from the medium. The histogram was calculated as described in the legend of Figure 4. The data represent the means ± standard deviations of RLU taken at 240 min from three independent experiments, and each sample was analyzed in duplicate.

To investigate whether the increase in the cytoplasmic Ca^2+^ level is due to the Ca^2+^ influx from the extracellular medium or due to the release from an internal store, we examined the effect of the rapid Ca^2+^ chelator BAPTA (1, 2-bis (o-aminophenoxy) ethane-N, N, N′, N′-tetraacetic acid). As shown in Figure 5A, the synergistic increase was inhibited by the addition of BAPTA in a dose-dependent manner, indicating that the increase in the cytoplasmic Ca^2+^ level is dependent on the influx across the Ca^2+^ channel that exists on the plasma membrane.

We have previously reported that the deletion of the *yam8* ^+^ or *cch1* ^+^ gene that encodes the putative subunit of a Ca^2+^ channel abolished the NaCl-induced activation of calcineurin [10]. This prompted us to investigate whether the synergistic increase is mediated by the Cch1-Yam8 channel complex. In single and double knockout cells of Cch1 and/or Yam8, NaCl plus FK506 failed to cause the synergistic increase in the cytoplasmic Ca^2+^ level (Figure 5B). These results suggest that the effect of FK506 is caused by the activation of the Cch1-Yam8 channel complex, and this is consistent with the report that in budding yeast calcineurin dephosphorylates and negatively regulates the Cch1-Yam8 channel complex [29].

### Knockout of the genes encoding protein kinase C-Pmk1 MAP kinase pathway abolished the synergistic increase in the cytoplasmic Ca^2+^ level

**Figure 6:**
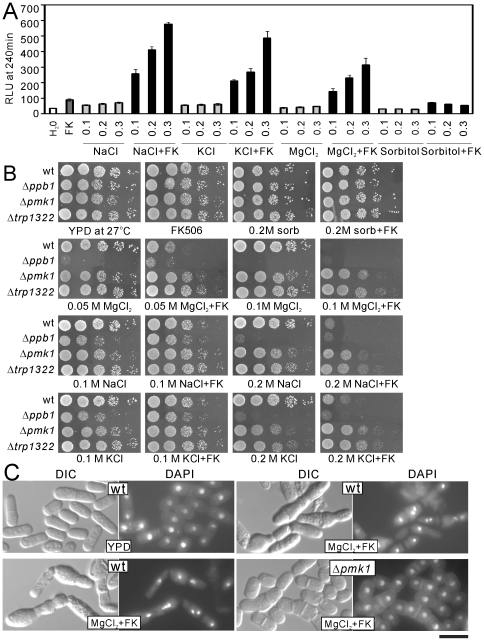
Salts plus FK506 caused a synergistic cytoplasmic Ca^2+^ increase and caused the cell death via an apoptotic process. (A) The addition of NaCl, KCl and MgCl_2_, but not sorbitol, caused the synergistic increase in the cytoplasmic Ca^2+^ level in the presence of FK506. The experiment was performed as described in Figure 4, except that prior to the addition of FK506, various concentrations of NaCl, KCl, MgCl_2_ and sorbitol, were added to the medium. (B) Effects of high salts and sorbitol on cell growth in the absence or presence of FK506. The wild-type (wt), Δ*ppb1*, Δ*pmk1*, or Δ*trp1322* cells were spotted onto the plates as indicated in serial 10-fold dilutions starting with cells (5 µl) grown at log phase with OD660 = 0.3, then incubated for 4 days at 27°C. (C) DAPI staining of wild-type and Δ*pmk1* cells. The wild-type and Δ*pmk1* cells were grown to exponential phase, then the cells were divided into two portions, one portion was cultured in YPD liquid, and the other portion was cultured in YPD liquid containing 0.15 M MgCl_2_ plus FK506 for 6 hours. Then the DAPI staining was performed as described in Materials and Methods. Bar, 10 µm.

It was also reported that the protein kinase C-MAP kinase pathway regulates the Cch1-Yam8 channel complex [29]. This prompted us to examine the cytoplasmic Ca^2+^ levels in the knockout cells of the *pmk1* ^+^ gene, encoding the MAP kinase. As shown in Figure 5C in Δ*pmk1* cells, the basal cytoplasmic Ca^2+^ level was extremely low and the synergistic Ca^2+^ increase was completely abolished. Consistent with our hypothesis that the persistently increased level of cytoplasmic Ca^2+^ causes cell death, the Δ*pmk1* cells grew well in the presence of FK506 plus high concentration of salts (Figure 6B, Δ*pmk1*). Knockout of the genes encoding MAPKK *pek1* ^+^ and MAPKKK *mkh1* ^+^, also completely abolished the synergistic increase and suppressed the cell death (data not shown).

### Overexpression of the constitutively active Pek1 MAPKK promotes Ca^2+^ influx via the Cch1-Yam8 channel complex

Pek1^DD^ is the constitutively active mutant of MAPKK, Pek1 [17]. To observe the effect of Pek1^DD^ overexpression on the cytoplasmic Ca^2+^ level, we constructed the pREP1-GST-Pek1^DD^ integrated cells as described in Materials and Methods. When Pek1^DD^ was overexpressed, NaCl plus FK506 caused an approximately 2-fold increase in RLU (Figure 5D, wt, lane 8 to 4). In contrast, the deletion of the *cch1* ^+^ gene totally abolished the synergistic increase of NaCl plus FK506 caused by Pek1^DD^ overexpression (Figure 5D, Δ*cch1*). The deletion of the *yam8* ^+^ gene also abolished the effect of Pek1^DD^ overexpression (data not shown). These results suggest that the calcineurin and the Pmk1 MAPK pathways antagonistically regulate the Ca^2+^ influx via the Cch1-Yam8 channel complex. Notably, when Pek1^DD^ was overexpressed, FK506 elicited a marked increase in RLU (Figure 5D, lane 7 to 3). We speculate that in wild-type cells even when Pek1^DD^ is overexpressed the dephosphorylation activity of calcineurin is higher than the phosphorylation activity of Pmk1, so that most of the Cch1-Yam8 channels are maintained in a closed state (Figure 5D, lane 5 and 6). Upon FK506 addition, however, the dephosphorylation activity of calcineurin is inhibited, so that most of the Cch1-Yam8 channels are maintained in the phosphorylated state, thus promoting the influx of calcium (Figure 5D, lane 7 and 8).

### The synergistic persistent increase in the cytoplasmic Ca^2+^ level causes cell death

In our previous study, we showed that the Δ*ppb1* cells are hypersensitive to NaCl, KCl or MgCl_2_ [30]. This prompted us to examine whether KCl, MgCl_2_ or sorbitol shows a synergistic effect with FK506 on the cytoplasmic Ca^2+^ level in wild-type cells. As shown in Figure 6A, NaCl, KCl and MgCl_2_, but not sorbitol, caused the synergistic increase in the cytoplasmic Ca^2+^ level in the presence of FK506, indicating that the synergistic slow and persistent increased level of cytoplasmic Ca^2+^ may cause cell death. Next, we examined the growth of wild-type and Δ*ppb1* cells in the presence of the salts alone and the salts plus FK506. The inhibitory effect on the cell growth showed good correlation with the increase in the cytoplasmic Ca^2+^ level caused by each agent (Figure 6B, wt and Δ*ppb1*). These results suggest that a persistently increased level of cytoplasmic Ca^2+^, but not Cl^−^, causes cell death.

To investigate whether the cell death is due to an apoptotic process or a nonspecific necrotic death, we stained the wild-type and Δ*pmk1* cells with DAPI to visualize the nuclei in the presence of MgCl_2_ plus FK506. Fluorescence microscopic imaging of DAPI-stained cells revealed nuclear fragmentation in wild-type cells treated with MgCl_2_ plus FK506, whereas the nuclei of Δ*pmk1* cells remained big, round and intact for the same time course of treatment (Figure 6C). These results suggest that the cell death is due to an apoptotic process.

Our previous results showed that high extracellular NaCl and KCl, but not MgCl_2_, caused the Cch1-Yam8-dependent activation of calcineurin. However, as described above, high extracellular MgCl_2_ caused a synergistic increase in the cytoplasmic Ca^2+^ level. Why MgCl_2_ increased the cytoplasmic Ca^2+^ level whereas it failed to activate calcineurin? We speculate that the elevation in intracellular Mg^2+^ may antagonize the activation of calcineurin by Ca^2+^, thus Mg^2+^ plus FK506 is more potent than either Na^+^ or K^+^ plus FK506 in the inhibition of the cell growth.

### The overexpression of Trp1322 suppressed the synergistic effect of NaCl plus FK506

**Figure 7:**
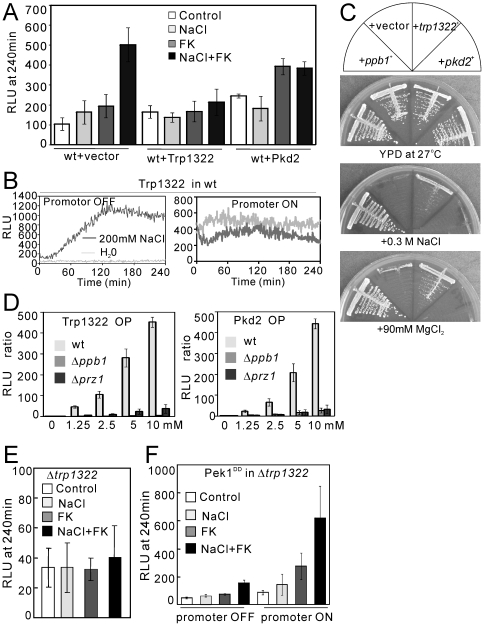
The role of Trp1322 in the regulation of calcium homeostasis in fission yeast. (A) The overexpression of Trp1322 and Pkd2 abolished the synergistic effect of NaCl plus FK506. The KP3028 cells were transformed with the control vector, pREP1-GFP-Trp1322 or pREP1-Pkd2-GFP, then were cultured as described in Figure 2A, and were assayed as described in Figure 4. The histogram was calculated as described in the legend of Figure 4. (B) When Trp1322 was overexpressed, the high basal CDRE-reporter activity was significantly decreased by the addition of NaCl. Note the marked activation of the reporter activity by the same stimuli in non-induced cells (Promoter OFF, left pannel). The KP2755 (*h^−^ leu1 arg1* 3×CDRE::luc(R2.2)::*arg1^+^*) cells were transformed with pREP1-GFP-Trp1322. The assay was performed as described in Figure 3B. The data shown are representative of multiple experiments. (C) The overexpression of *trp1322* ^+^ gene suppressed the salts sensitivity of Δ*ppb1* cells. The Δ*ppb1* cells transformed with the control vector, pREP1-GFP-Trp1322 or pREP1-Pkd2-GFP were streaked onto the plates as indicated and then incubated for 4 days at 27°C. (D) The effect of the overexpression of Pkd2 or Trp1322 on the cytosolic Ca^2+^ level upon the addition of extracellular calcium in Δ*ppb1* and Δ*prz1* cells. The KP3028 (wild-type), KP3750 (Δ*ppb1*) or KP3688 (Δ*prz1*) cells transformed with the control vector, pREP1-GFP-Trp1322 or pREP1-Pkd2-GFP, respectively were cultured and assayed as described in Figure 2A. The RLU ratio was determined by dividing the peak RLU at 0 mM CaCl_2_ by the peak RLU at 1.25, 2.5, 5, and 10 mM CaCl_2_, respectively. The data represent the means ± standard deviations of RLU ratio from three independent experiments, and each sample was analyzed in duplicate. (E) The deletion of *trp1322* ^+^ gene suppressed the synergistic effect of NaCl plus FK506. The Δ*trp1322* cells harboring pKB6892 were cultured and assayed as described in Figure 4. The histogram was calculated as described in the legend of Figure 4. The data represent the means ± standard deviations of RLU taken at 240 min from three independent experiments, and each sample was analyzed in duplicate. (F) Overexpression of the constitutively active Pek1 MAPKK also stimulates Ca^2+^ influx in Δ*trp1322* cells. The Δ*trp1322* cells integrated with the chromosomal pREP1-GST-Pek1^DD^ were transformed with pKB6892, and the transformants were cultured in EMM with the addition of 4 µM thiamine for 12 hours. Then the assay was performed as described in the legend of Figure 5D. The histogram was calculated as described in the legend of Figure 4. The data represent the means ± standard deviations of RLU taken at 240 min from three independent experiments, and each sample was analyzed in duplicate.

We then examined the effect of NaCl plus FK506 on the cells overexpressing Trp1322 or Pkd2 in wild-type cells. Overexpression of these two TRP channels showed high basal cytoplasmic Ca^2+^ levels in the absence of NaCl or FK506 (Figure 7A, control). In cells overexpressing Trp1322 or Pkd2 when NaCl alone was added to the medium, the high basal cytoplasmic Ca^2+^ level was decreased, while in cells harboring the vector the cytoplasmic Ca^2+^ level was markedly elevated (Figure 7A, +NaCl). Consistently, in wild-type cells when Trp1322 was overexpressed the high basal CDRE-reporter activity was significantly lowered by the addition of NaCl (Figure 7B, promoter ON). Surprisingly, when the cells overexpressing Trp1322 were treated with NaCl plus FK506, no marked increase in the cytoplasmic Ca^2+^ level was observed (Figure 7A, wt+Trp1322, NaCl+FK). These results suggest that the overexpression of Trp1322 may inhibit the Ca^2+^ influx via the Cch1-Yam8 channel complex. Consistently in Δ*ppb1* cells, the overexpression of Trp1322 significantly suppressed the NaCl and MgCl_2_ sensitivity (Figure 7C).

Then we examined the effect of the overexpression of Pkd2 or Trp132 on the cytoplasmic Ca^2+^ levels upon the addition of extracellular calcium in Δ*ppb1* cells. As shown in Figure 7D, in wild-type cells the overexpression of Trp1322 induced an approximately 420–470 fold increase in the cytoplasmic Ca^2+^ levels by the extracellularly added 10 mM CaCl_2_ as compared with the control (Figure 7D, left panel). This burst-like increase was completely abolished in Δ*ppb1* cells and was dramatically decreased in Δ*prz1* cells (Figure 7D, left panel). The effect of Pkd2 overexpression also dramatically decreased in Δ*ppb1* cells and Δ*prz1* cells as compared with that in wild-type cells (Figure 7D, right panel).

### Deletion of the *trp1322* ^+^ gene suppressed the synergistic effect of NaCl plus FK506

Our results also showed that in Δ*trp1322* cells the basal cytoplasmic Ca^2+^ level was extremely low and the synergistic increase in the cytoplasmic Ca^2+^ level caused by NaCl plus FK506 was markedly suppressed (Figure 7E). Consistently, deletion of the *trp1322* ^+^ gene significantly suppressed the cell death caused by salts plus FK506 (Figure 6B, Δ*trp1322*). Thus, both the overexpression and deletion of the *trp1322* ^+^ gene antagonize the synergistic effect of NaCl plus FK506 on the Ca^2+^ influx via the Cch1-Yam8 channel complex. Deletion of the *trp663* ^+^ gene had no effect on the synergistic Ca^2+^ increase (data not shown).

To further investigate the relationship between the Trp1322 channel and the Pmk1 MAPK pathway, we monitored the effect of the overexpression of Pek1^DD^, a constitutively active mutant of MAPKK [17], in Δ*trp1322* cells. Overexpression of Pek1^DD^ recovered the synergistic effect of NaCl plus FK506 in Δ*trp1322* cells (Figure 7F). These results suggest that deletion of the *trp1322* ^+^ gene decreases the cytoplasmic Ca^2^+ level, but does not abolish the opening of the Cch1-Yam8 channel complex by the activation of the Pmk1 MAPK pathway.

## Discussion

In the present study, we have developed a highly sensitive assay for the intracellular Ca^2+^ level using GFP-19-AEQ and have successfully monitored the Ca^2+^ level in living fission yeast cells for a long period of up to 4–6 hours. This enabled us to find a novel role of calcineurin in the regulation of the cytoplasmic Ca^2+^ concentration.

### The resting cytoplasmic Ca^2+^ level and the effect of extracellularly added CaCl_2_

In the present study, a first ever monitoring of the molar concentration of the cytosolic Ca^2+^ level in living fission yeast cells was conducted. The results showed that the resting cytosolic Ca^2+^ concentration varies from 100∼200 nM, and stimulation by 100 mM CaCl_2_ induced an approximately 6∼10 fold burst-like increase at the peak of the burst. Our data are consistent with the resting and activated Ca^2+^ level in mammalian and budding yeast [31]–[33].

The dose-dependent rise in the cytosolic Ca^2+^ level (Figure 2) is likely to be explained by the influx of extracellular Ca^2+^ through the TRP channels. Consistently, this response is markedly enhanced by overexpression of the TRP channel homologues, Trp1322 and Pkd2. Interestingly, the overexpression of another TRP channel TRP663, which showed the most significant plasma membrane localization, failed to elicit an increase in the cytosolic Ca^2+^ level, suggesting that not all TRP channels will lead to an increase in the cytosolic Ca^2+^ level. In addition, we overexpressed the *cch1* ^+^ and *yam8* ^+^ genes as described in Methods S1, and the results showed that the co-overexpression of Yam8 and Cch1 did not elicit a cytosolic Ca^2+^ increase (Figure S1). Consistently, the Ca^2+^ signal observed in Figure 2 and 3A was also observed in Δ*yam8* and Δ*cch1* (data not shown). Altogether our results indicate that the overexpression of some calcium channels (Trp1322, Pkd2) mediate a rise in the cytosolic Ca^2+^ level whereas other channels (Trp663, Cch1-Yam8) do not. Also, the release from the intracellular organelles (ER, Golgi or vacuoles) may play an important role in the cytosolic Ca^2+^ increase.

The dose-dependent rise in the cytosolic Ca^2+^ level (Figure 2) showed a burst-like nature (i.e. initial sharp peak followed by a slow decay). This burst-like nature is likely to be explained by the Ca^2+^ diffusing through the two TRP channels to raise the cytosolic Ca^2+^ level which in turn activates Ca^2+^ sensor protein, thus resulting in the closing of the channels. In mammalian cells, it is well known that Ca^2+^ channels exhibit a Ca^2+^-induced inactivation mediated by calmodulin [25], [34], [35] or other calcium sensor proteins that are conserved in fission yeast [26]. The rapidly decaying signal in Figure 2 is likely the result of the channel inactivation caused by a Ca^2+^ sensor protein. However, in both *cam1-1* mutants (Figure 2D) and Δ*ncs1* cells (data not shown), the burst-like peak was also observed in wild-type cells.

Palmer et al. speculated that *pkd2* ^+^ may encode a mechanosensitive ion channel based on the observation that modulators of mechanosensitive ion channels such as lysophosphatidylinositol or gadolinium affected the growth of the Pkd2-depleted strain [7], [8]. In the present study, however, the use of agents such as high salts or sorbitol that may stimulate the mechanosensitive Cch1-Yam8 channel complex failed to open the Pkd2 or Trp1322 channels. Because extracellularly added CaCl_2_ also produced a dose-dependent rise in the cytoplasmic Ca^2+^ level, we suggest that Trp1322 and Pkd2 are Ca^2+^ permeable, but not mechanosensitive, channels. Another TRP homologue, Trp663, on the other hand does not seem to be involved in this process, although Trp663 has a higher amino acid sequence identity to Pkd2, as compared with that of Trp1322.

Our results also showed that upon the addition of CaCl_2_ the effect of Trp1322 and Pkd2 overexpression is totally abolished in Δ*ppb1* cells, and is partially abolished in Δ*prz1* cells (Figure 7D). In Δ*pmr1* cells (Figure 3D), Δ*pmc1* or Δ*vcx1* cells (data not shown), the burst-like peaks were observed with an increased magnitude of the peak. Furthermore, the FK506 treatment 1 hour before the CaCl_2_ stimulation failed to suppress the burst-like peak while the FK506 treatment 24 hours before CaCl_2_ stimulation completely abolished the peak (data not shown). Altogether these results suggest that the effect of calcineurin gene deletion is mainly due to the long-term inhibition of Prz1-dependent transcription, but not due to the inhibition of the direct TRP dephosphorylation by calcineurin.

### The antagonistic regulation of the Pmk1 MAPK pathway and calcineurin pathway modulates the cytosolic Ca^2+^ concentration

Why does NaCl plus FK506 cause a slow and persistent increase in the cytoplasmic Ca^2+^ level in fission yeast cells? What is the molecular mechanism that generates the synergistic Ca^2+^ signal? How do NaCl and FK506 work together? In Δ*prz1* cells, the synergistic increase in the cytoplasmic Ca^2+^ level was clearly observed suggesting that the regulation of calcineurin on Cch1-Yam8 is not due to the transcriptional regulation by Prz1. The deletion of the *pmk1* ^+^ gene markedly suppressed the Ca^2+^ influx, and overexpression of the constitutively active Pek1 MAPKK enhanced the Ca^2+^ influx, indicating that Pmk1 MAPK pathway may have a positive regulation on the Cch1-Yam8 channels.

Furthermore, the following results are presented. First, similar to Δ*pmk1* cells, the basal cytoplasmic Ca^2+^ level in Δ*trp1322* cells was extremely low, and the synergistic increase in the cytoplasmic Ca^2+^ level caused by NaCl plus FK506 was markedly suppressed. Second, similar to the deletion of *pmk1*, the deletion of the *trp1322* ^+^ gene significantly suppressed the cell death caused by salts plus FK506. Third, similar to MAPKK Pek1, the overexpression of Trp1322 suppressed the salts-sensitive phenotype of Δ*ppb1* cells. Fourth, in Δ*trp1322* cells, the overexpression the constitutively active Pek1^DD^ also elicit the synergistic effect of NaCl plus FK506 (Figure 7F). Altogether, these results indicate that Trp1322 may crosstalk with Pmk1 MAPK pathway, thus indirectly affecting the Cch1-Yam8 complex upon NaCl plus FK506 stimulation.

**Figure 8:**
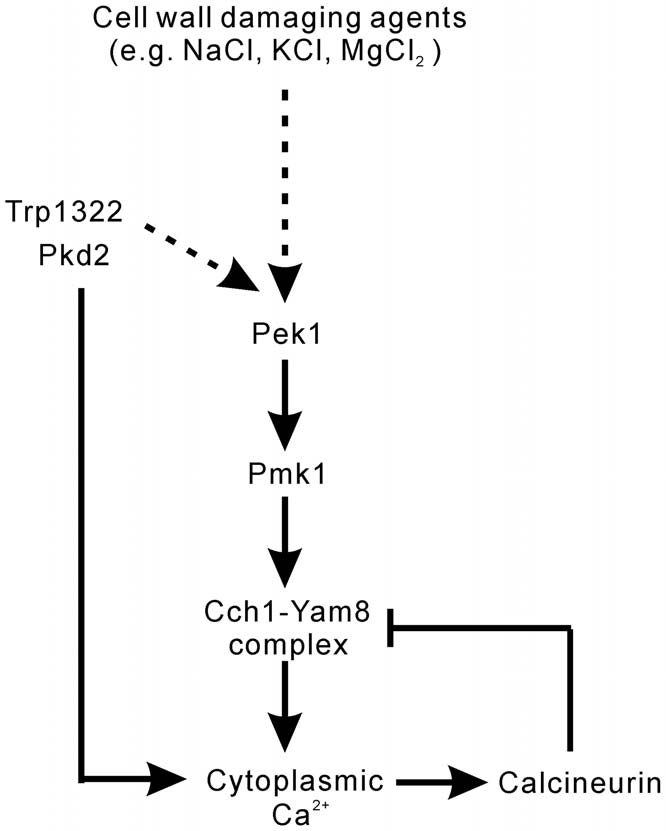
A cartoon figure illustrating a molecular mechanism of Ca^2+^ signaling. The cell wall damaging agents (e.g. NaCl, KCl, MgCl_2_) activate the cell wall integrity MAPK Pmk1, then the activated Pmk1 phosphorylates Cch1, resulting in the opening of the Cch1-Yam8 channel and in Ca^2+^ influx. In the absence of FK506, the increase in the cytoplasmic Ca^2+^ level activates calcineurin, which in turn dephosphorylates Cch1, resulting in the closing of the channel. In the presence of FK506, calcineurin is inhibited, thus resulting in the persistent opening of the Cch1-Yam8 channels. Trp1322 and Pkd2 directly regulates calcium influx, and also may crosstalk with other components of the Pmk1 MAPK pathway upstream of Pek1, thus indirectly affecting the Cch1-Yam8 complex upon NaCl plus FK506 stimulation.

It has been reported that in budding yeast calcineurin can directly dephosphorylate Cch1, resulting in the inhibition of the Cch1-Mid1 channel [29]. In the present study, we summarize our hypothesis in Figure 8. As shown in the figure, the NaCl addition damages the cell wall and activates the cell wall integrity MAPK Pmk1, and the activated Pmk1 phosphorylates Cch1, resulting in the opening of the Cch1-Yam8 channel and leading to a Ca^2+^ influx. In the absence of FK506, the cytosolic Ca^2+^ increase activates calcineurin, which in turn dephosphorylates Cch1, resulting in the closing of the channel. In the presence of FK506, FK506 inhibits calcineurin, thus resulting in the persistent opening of the Cch1-Yam8 channels.

Our present study indicates that NaCl plus FK506 caused a slow and persistent increase in the cytoplasmic Ca^2+^ level in fission yeast cells. In addition, KCl plus FK506 and MgCl_2_ plus FK506 also caused a synergistic increase in the cytoplasmic Ca^2+^ level similar to that caused by NaCl plus FK506. Furthermore, similar to Δ*ppb1* cells, the wild-type cells failed to grow in the presence of NaCl/KCl/MgCl_2_ plus FK506. The Δ*pmk1* cells, on the other hand, displayed an extremely low cytoplasmic Ca^2+^ level, and grew in the presence of NaCl/KCl/MgCl_2_ plus FK506. MgCl_2_ increased the cytoplasmic Ca^2+^ level whereas it failed to activate calcineurin, thus Mg^2+^ plus FK506 is more potent than either Na^+^ or K^+^ plus FK506 in the inhibition of the cell growth. Therefore, as described above, we hypothesize that a persistently increased level of cytoplasmic Ca^2+^, but not Cl^−^, causes cell death when wild-type cells are treated with these salts plus FK506, or when Δ*ppb1* cells are treated with these salts. Presumably, the cell wall defects caused by high salts stimulate the Rho/Pck/MAPK cell wall integrity pathway [36] and this, in turn, triggers a Ca^2+^ influx through the Cch1-Yam8 channel complex which is negatively regulated by calcineurin (Figure 8). Interestingly, high sorbitol did not cause the synergistic increase in the Ca^2+^ level in the presence of FK506, suggesting that high salts and sorbitol affect the cell wall integrity signaling via different mechanisms. Altogether, our present study indicates that the TRP and Cch1-Yam8 channels play key roles in the regulation of cytoplasmic Ca^2+^ in fission yeast. These two channels are also involved in the cell wall integrity signaling and are coordinately and antagonistically regulated by the Pmk1 MAPK pathway and the calcineurin pathway. Further studies are needed to elucidate the gating mechanism of these channels and its physiological relevance to cell function.

## Supporting Information

Figure S1 **Co-overexpression of Cch1 and Yam8 channel complex failed to significantly increase the CaCl_2_-induced burst-like peak.** The KP5088 cells (*h^−^ leu1-32 ura4-294 arg1-1 pREP1-Yam8-GFP*::*ura4^+^ pREP1-GFP-19aa-AEQ*::*arg1^+^*) were transformed with a control vector and pREP1-Cch1-GFP respectively. The transformants were grown to exponential phase in the absence of thiamine for 36 hours to co-overexpress GFP-19-AEQ, Yam8 and Cch1. The monitoring of the cytoplasmic Ca^2+^ level was performed as described in Figure 2A. The data shown are representative of multiple experiments.
(TIF)

Methods S1 **Overexpression of the** ***cch1*** **^+^ and** ***yam8*** **^+^ genes.**
(DOCX)
