# Supplementary figures and images for: Transient Receptor Potential (TRP) and Cch1-Yam8 Channels Play Key Roles in the Regulation of Cytoplasmic Ca2+ in Fission Yeast
Source: PLoS One. 2011 Jul 19;6(7):e22421. doi: 10.1371/journal.pone.0022421 (PMC3139647; doi:10.1371/journal.pone.0022421)

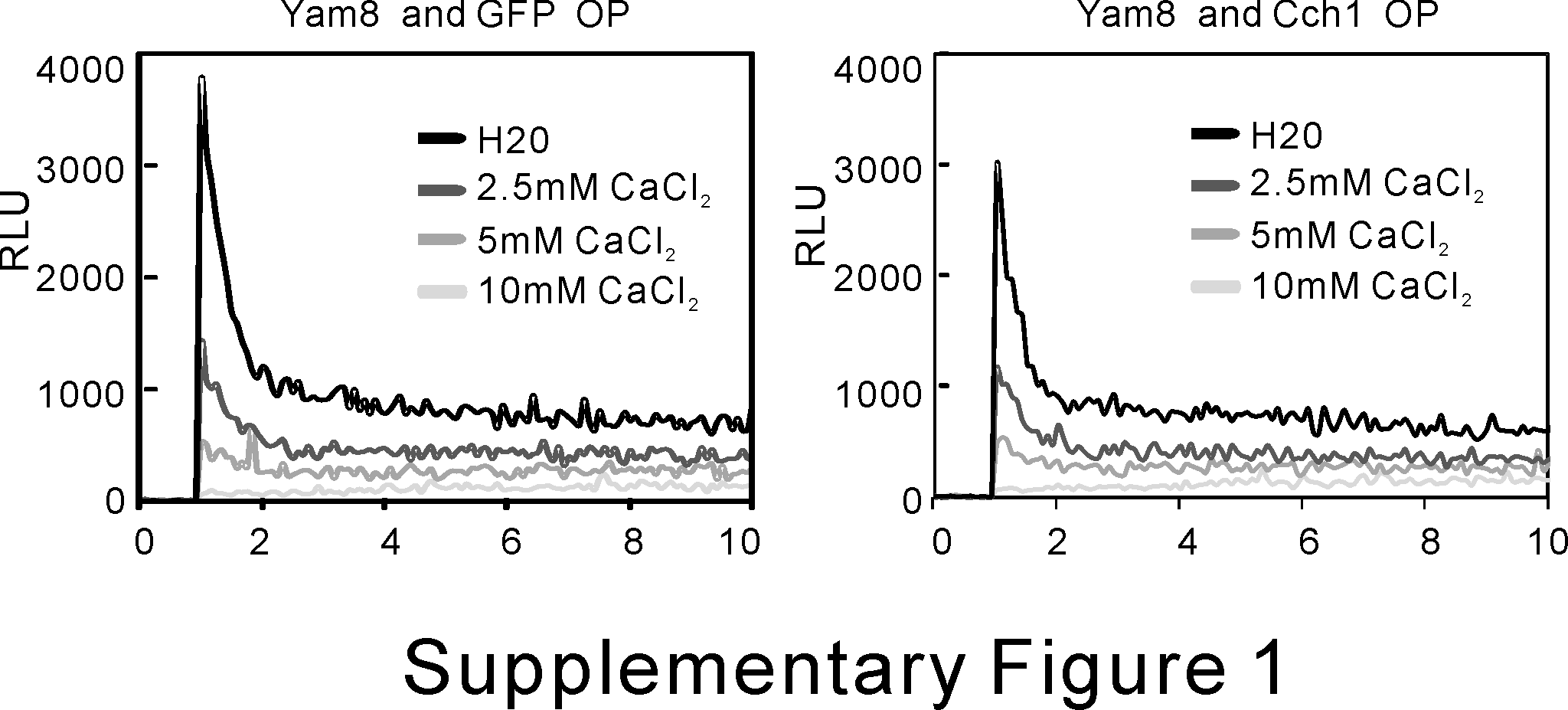

Supplement: Figure S1 — Co-overexpression of Cch1 and Yam8 channel complex failed to significantly increase the CaCl2-induced burst-like peak. The KP5088 cells (h− leu1-32 ura4-294 arg1-1 pREP1-Yam8-GFP::ura4+ pREP1-GFP-19aa-AEQ::arg1+) were transformed with a control vector and pREP1-Cch1-GFP respectively. The transformants were grown to exponential phase in the absence of thiamine for 36 hours to co-overexpress GFP-19-AEQ, Yam8 and Cch1. The monitoring of the cytoplasmic Ca2+ level was performed as described in Figure 2A. The data shown are representative of multiple experiments. (TIF) [file pone.0022421.s001.tif]
