# Supplementary material for: Transient Receptor Potential (TRP) and Cch1-Yam8 Channels Play Key Roles in the Regulation of Cytoplasmic Ca2+ in Fission Yeast
Source: PLoS One. 2011 Jul 19;6(7):e22421. doi: 10.1371/journal.pone.0022421 (PMC3139647; doi:10.1371/journal.pone.0022421)
Supplement: Methods S1 — Overexpression of the cch1 + and yam8 + genes. (DOCX) [file pone.0022421.s002.docx]

Supplementary Materials and Methods

Overexpression of the *cch1*^+^ and *yam8*^+^ genes

The *cch1*^+^ and *yam8*^+^ genes were amplified by PCR using the genomic DNA of wild-type cells as a template. To express Cch1-GFP, the complete ORF of *cch1^+^* was amplified using the sense primer 5’-CGC GGA TCC ATG TCA TCA AGC TCA AAC TCA GAT CC-3’, and the antisense primer 5’-CGC GGA TCC GCT CCT TTA AAT GAA TCC CGA TC-3’. The complete ORF of *yam8^+^* was ligated to the C-terminus of the GFP carrying the S65T mutation [1].

To express Yam8-GFP, the complete ORF of *yam8^+^* was amplified using the sense primer 5’-GGA AGA TCT ATG TTT TTT TTT AGC ACC C-3’, and the antisense primer 5’-GGA AGA TCT GCC TCG ACA AAC AAA ATC CAG-3’. The complete ORF of *yam8^+^* was ligated to the C-terminus of the GFP carrying the S65T mutation [1]. To obtain the chromosome-borne pREP1-Yam8-GFP, the *Pst*I/*Sac*I fragment containing pREP1-Yam8-GFP was subcloned into the vector containing the *ura4*^+^ marker and was integrated into the chromosome at the *ura4*^+^ gene locus of KP1248 (h^+^ *leu1-32 ura4-294*) as described [2,3].

Supplementary References

1. Heim R, Cubitt AB, Tsien RY (1995) Improved green fluorescence. Nature 373: 663-664

2. Cheng H, Sugiura R, Wu W, Fujita M, Lu Y, et al. (2002) Role of the Rab GTP-binding protein Ypt3 in the fission yeast exocytic pathway and its connection to calcineurin function. Mol Biol Cell 13: 2963-2976

3. Kita A, Sugiura R, Shoji H, He Y, Deng L, et al. (2004) Loss of Apm1, the micro1 subunit of the clathrin-associated adaptor-protein-1 complex, causes distinct phenotypes and synthetic lethality with calcineurin deletion in fission yeast. Mol Biol Cell 15: 2920-2931
